# Comparison of different mechanical chest compression devices in the alpine rescue setting: a randomized triple crossover experiment

**DOI:** 10.1186/s13049-021-00899-x

**Published:** 2021-06-29

**Authors:** Egger Alexander, Tscherny Katharina, Fuhrmann Verena, Grafeneder Jürgen, Niederer Maximilian, Kienbacher Calvin, Schachner Andreas, Schreiber Wolfgang, Herkner Harald, Roth Dominik

**Affiliations:** 1 Mountain Rescue Service Austria, Schelleingasse 26/2/2, 1040 Wien, Austria; 2 Department of Anaesthesiology and Intensive Care Medicine, Hospital Scheibbs, Eisenwurzenstraße 26, 3270, Scheibbs, Austria; 3 Department of Emergency Medicine, Medical University of Vienna, Spitalgasse 23, 1090 Wien, Austria; 4 Department of Clinical Pharmacology, Medical University of Vienna, Spitalgasse 23, 1090 Wien, Austria

**Keywords:** Mechanical chest compression, Alpine rescue mission, Out-of-hospital cardiac arrest, Hypothermic cardiac arrest

## Abstract

**Background:**

Cardiopulmonary resuscitation in mountain environment is challenging. Continuous chest compressions during transport or hoist rescue are almost impossible without mechanical chest compression devices. Current evidence is predominantly based on studies conducted by urbane ambulance service. Therefore, we aimed to investigate the feasibility of continuous mechanical chest compression during alpine terrestrial transport using three different devices.

**Methods:**

Randomized triple crossover prospective study in an alpine environment. Nineteen teams of the Austrian Mountain Rescue Service trained according to current ERC guidelines performed three runs each of a standardised alpine rescue-scenario, using three different devices for mechanical chest compression. Quality of CPR, hands-off-time and displacement of devices were measured.

**Results:**

The primary outcome of performed work (defined as number of chest compressions x compression depth) was 66,062 mm (2832) with Corpuls CPR, 65,877 mm (6163) with Physio-Control LUCAS 3 and 40,177 mm (4396) with Schiller Easy Pulse. The difference both between LUCAS 3 and Easy Pulse (Δ 25,700; 95% confidence interval 21,118 – 30,282) and between Corpuls CPR and Easy Pulse (Δ 25,885; 23,590 – 28,181) was significant. No relevant differences were found regarding secondary outcomes.

**Conclusion:**

Mechanical chest compression devices provide a viable option in the alpine setting. For two out of three devices (Corpuls CPR and LUCAS 3) we found adequate quality of CPR. Those devices also maintained a correct placement of the piston even during challenging terrestrial transport. Adequate hands-off-times and correct placement could be achieved even by less trained personnel.

## Background

In 2020, 261 people died in the Austrian alps. Contrary to popular believe, only about 4 % of them died because of avalanche burial, whereas 22% died due to non-traumatic cardiac arrest. The majority of patients with non-traumatic cardiac arrest were aged between 50 and 80 years [1], reflecting an increasingly older population visiting the mountain environment.

High-quality chest compressions and early defibrillations are the cornerstones of successful cardiopulmonary resuscitation (CPR). This is true both in urban and mountain environment. Transportation under continuous chest compressions is generally recommended in case of potentially reversible causes that can only be adequately treated in a hospital. This is true for myocardial infarction, but also for special circumstances often encountered in the alpine environment, such as hypothermia [2].

However, transportation under continuous chest compression is a special challenge for terrestrial and helicopter rescue crews [3]. Continuous manual chest compression is impossible during a fixed rope rescue or hoist rescue operation in helicopter emergency medical service (HEMS). In other cases, continuous manual chest compression could not be performed in high quality without severe risk for rescue personnel, like in challenging terrain with potential falling hazard. Unacceptable risk to rescuer or rescuer exhaustion are reasons for termination of resuscitation, even if there is a potentially reversible underlying cause of cardiac arrest [4]. Furthermore, recent literature shows a significant decrease in quality of manual chest compression of experienced subjects after physical strain in high altitude [5]. There is also an increase in the rigidity of the thorax in case of profound hypothermia (core body temperature below 20 °C) [6]. This could further aggravate the exhaustion of rescuers.

Current available devices for mechanical chest compression might also be suitable for the alpine rescue mission. The mountain environment imposes many challenges which are not present in the settings of in-hospital emergency medicine and emergency medical service (EMS).

The need for manual transportation to scene, prolonged transportation time without any possibility of recharging and lack of continuous control of correct piston placement as well as extreme climatic conditions are some of these special circumstances. The usage of mechanical chest compression devices nevertheless offers alpine rescue crews’ new possibilities in high quality CPR.

Most of the research on mechanical chest compression devices has been performed in the urban or flat rural setting. Whereas Havel et al. [7] did not find significant differences in quality of manual chest compressions during transportation in an ambulance vehicle or helicopter transport compared to on scene, Putzer et al. [8] were able to detect a significant superiority of mechanical chest compression during helicopter transport. This finding was also supported by a study of Gaessler et al. [9] in 2015.

A few case reports support the practicability and possible good neurological outcome after continuous chest compressions in an alpine rescue setting, or transportation of deep hypothermic patients [2, 10, 11]. Mechanical CPR was however only performed on site in one of them [10], and was started at the hospital in another [11]. There is currently only one controlled study on modes of CPR during transportation in the alpine environment. Thomassen et al. [12] could demonstrate that it was possible to maintain high-quality chest compression both manual and mechanical in such a setting. The applicability of these findings is however limited by the setting of transportation in a sledge attached to a snowmobile on a very flat slope with a descent of only 18%. A sledge in combination with a snowmobile is a preferred option of transport in snow covered moderate steep area, like the polar cap, some ski-slopes or glaciers. It offers fast transport by a two-man rescue team. In many regions, e.g. the Alps of Central Europe, the majority of alpine rescue missions however takes place on steep hiking trails, where transportation using alpine stretchers is necessary. Sledges cannot be used here. This results in longer duration of transport and need for more manpower. Most importantly, the mechanical impact of a transport on a stretcher over rough terrain is usually much higher than the rather smooth ride on a sledge.

We hence aimed to investigate feasibility and quality of mechanical chest compression under real alpine conditions.

## Methods

This is a randomized triple crossover prospective study in an alpine environment. Two-person teams completed a standardized alpine rescue scenario three times, using three different battery-powered mechanical chest compression devices in a randomized order. The study was approved by the ethical review board of the Lower Austrian government (GS1-EK-1/197–2020).

### Study subjects

A total of 38 members of the Austrian mountain rescue service at least 18 years old participated in the study. Participants were randomized into 19 two-person teams. Each team consisted of one trained emergency medical technician (EMT) and one team member with regular training in Basic Life Support (BLS) according to the current ESC guidelines. This reflects the real-life composition of mountain rescue service teams, where only a limited number of team members have EMT training.

### Study setting

**Fig. 1:**
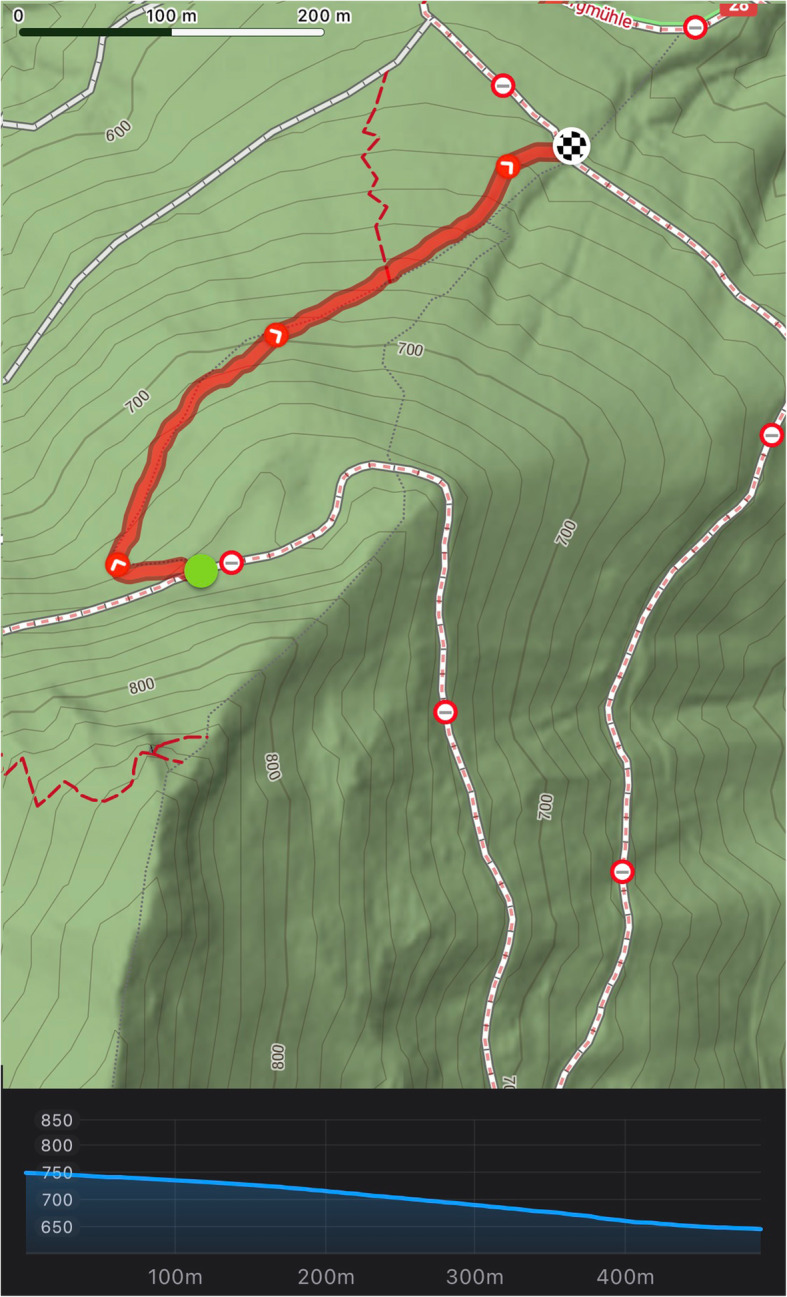
Map of hiking trail

This study was performed in alpine environment (Gaming, Lower Austria, Austria). The starting point was at 770 m above sea level, the arrival point at 630 m above sea level, the total distance was 490 m, the average decline was 22%, the maximum decline 38%. The study scenario track was situated on a hiking trail in rough terrain (see Fig. 1).

### Intervention and measurement

After informed consent, participants received two hours of hands-on instructions on the use of the three different chest devices (see below), two weeks before the study day.

Training focused on correct placement of the device and minimizing hands-of-time.

On the study day, participants performed three runs of a standardised alpine rescue-scenario using the three different devices. Order of devices was randomized for each team.

The non-EMT in the team started manual compression-only resuscitation on a manikin (Laerdal Q-CPR Full Body (weight 24 kg), Laerdal Medical AS, Stavanger, Norway), while the EMT prepared and applied the mechanical chest compression device. A member of the study-staff marked the initial position of the piston of the chest compression device using a felt pen.

After application and starting of the mechanical chest compression device in continuous mode, the manikin was transferred to a stretcher designed for alpine use (Tyromont Gebirgstrage Light, Tyromont Alpin Technik GmbH, Thaur, Austria), immobilised in a vacuum-mattress (RedVac VM01, medida GmbH & Co. KG, Stockstadt, Germany) and transport was begun. For the sake of simplicity, no ventilation was performed as part of the study. Average transportation time on the trail was measured to be 8 min beforehand.

### Devices

We compared Corpuls CPR (GS Elektromedizinische Geräte G. Stemple GmbH, Kaufering, Germany), LUCAS 3 (Physio-Control, Redmond, USA) and Easy Pulse (Schiller Medizintechnik GMBH, Feldkirchen, Germany) devices. We also aimed to include the AutoPulse (ZOLL Medical Corporation, Chelmsford, USA) device into our study, but the company decided to not provide a device for the trial because of the “unique” chest compression technology that does not reproduce current ERC guidelines in the setting of manikin study.

All devices are driven by rechargeable battery. Under normal conditions, manufacturers guarantee a working time of 45–90 min. Each of them had a second battery in case.

**Fig. 2:**
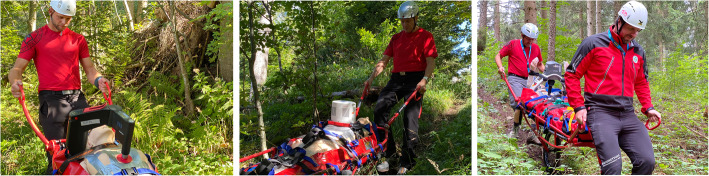
Devices used in the study: Corpuls CPR (left), Schiller Easy Pulse (middle), PhysioControl LUCAS 3 (right)

In addition to the stretcher used in the study, all devices were also tested for compatibility with the “Tyromont” rescuebag “Christophorus Evo” (Tyromont Alpin Technik GmbH, Bert-Köllensperger-Str. 6, A-6065 Thaur) to prove possibility of fixed rope rescue or hoist rescue operations in HEMS. Technical information for all devices, including the AutoPulse, is listed in Table 1. Depictions of all devices are shown in Fig. 2.

**Table 1:** Technical data of chest compression devices

	Corpuls CPR	Schiller Easy Pulse	PhysioControl LUCAS 3	ZOLL AutoPulse
**Weight (device)**	5.5 kg	3.5 kg	8 kg	10.6 kg
**Weight (including manikin)**	29.5 kg	27.5 kg	32 kg	34.6 kg
**Width**	43 cm	up to 40 cm	52 cm	44.7 cm
**Compressions per minute**	80 to 120/min	100/min	102 ± 2/min	80 ± 5/min
**Battery capacity**	90 min	45 min	45 min	30 min
**Temperature range**	−20 to 45 °C	-20 to 40 °C	-20 to 40 °C	0 to 40 °C
**Fits into Tyromont rescuebag**	+	+	+	Not tested
**Fits into Tyromont alpine stretcher**	+ (with scoopboard)	+	+	Not tested

#### Corpuls CPR

This is a piston-based compression device with the option of three different back plates with different forms. For the manikin study, we used the “Recboard” type back plate. The manufacturer also offers a special ring, to be placed on the patient’s thorax, for fixing the patient on the back plate. This is the only device with the option to change both compression depth (from 2 to 6 cm) and rate (from 80 to 120 compressions per minute). For this study we selected a compression depth of 5.5 cm, and 110 compressions per minute.

#### Physio-control LUCAS 3

This is also a piston-based compression device with two arms in lateral position. These two arms are being fixed on a universal backplate. The device uses a neckband against cranio-caudal dislocation. There are two different modes (30:2 or continuously with 102 +/− 2 compressions per minute). For the study setting, we used the continuous mode.

#### Schiller easy pulse

This device is a combination between piston and compression band, allowing “circulating” thorax compressions. The device is placed on the patient’s thorax and fixed with four straps on a back plate. The device uses additional shoulder straps against cranio-caudal dislocation. There are two different modes (30:2 and continuously with 100 compressions per minute).

For the study setting, we used the continuous mode.

### Outcomes

The primary outcome was performed work (number of chest compression x compression depth), scaled for descent time. Secondary outcomes included hands-off-time, relative proportion of effective chest-compressions, mean compression depth, mean compression rate, and deviation of the position of the piston over time. We also asked participants to rank the devices according to their personal preferences.

All measures of CPR-quality (see Analysis below) were automatically measured by the manikin and exported to Microsoft Excel (Microsoft Corp., Redmond, USA) afterwards. At the end of the track, a member of the study-staff measured movement of the compression piston from the marked position using a measuring tape.

### Statistical analysis

Sample size calculation were based on the primary outcome of performed work (number of chest compression x compression depth). Based on previous studies on CPR in difficult settings, such as aboard an aircraft or helicopter [8, 13], we expected performed work over 8 min to be 40,000 (standard deviation SD 4800) mm. To detect a meaningful difference of 10% between devices for this outcome we would have needed to include 18 teams (probability for error of 1st kind 5%, power 80%). To allow for the study design, we planned to include a total of 20 teams. Due to one participant’s lack of availability, we finally included 19 teams into the study.

We tabulated outcomes by device and calculated absolute differences with robust 95% confidence intervals using a linear random-effects regression model with the device as an independent variable. To verify successful randomization, we also performed additional analysis incorporating randomization sequence as a factor variable.

We used Stata 16MP (Stata Corp, College Station, USA) for all analyses. Generally, a two-sided *p*-value less than 0.05 was considered statistically significant.

## Results

### Characteristics of study subjects

A total of 38 participants in 19 teams were included in the study. Mean age of participants was 36 years (SD 12), mean body mass index was 26 (SD 4), they had an average of 9 years (SD 6) of service, and three (8%) participants were female. All subjects were able to complete the scenarios as planned.

### Main results

The average time taken for the descent was 11.8 min (SD 2) when using Corpuls CPR, 13.0 min (SD 2.3) for Physio-Control LUCAS 3 and 12.1 min (SD 1.9) for Schiller Easy Pulse. Main outcome was scaled to a duration of 12 min for all devices.

**Fig. 3:**
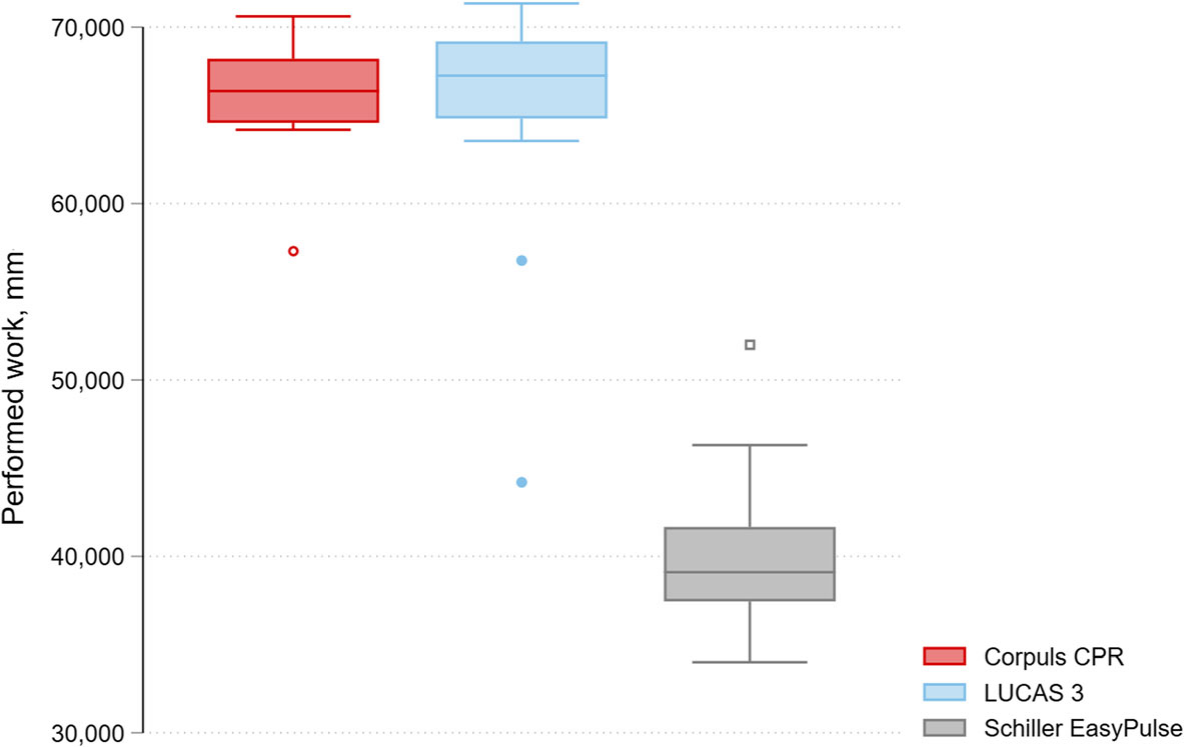
Main outcome (performed work) by device

The main outcome of performed work was 66,062 mm (SD 2832) with Corpuls CPR, 65,877 mm (6163) with LUCAS 3, and 40,177 mm (4396) with Easy Pulse, see Fig. 3.

There was a significant difference between LUCAS 3 and Easy Pulse (25,700; 95% confidence interval 21,118 to 30,282 mm). There was also a significant difference between Corpuls CPR and Easy Pulse (25,885; 95% CI 23,590 to 28,181 mm). There was no significant difference between LUCAS 3 and Corpuls CPR (185; 95%CI − 3346 to 3716 mm).

### Secondary outcomes

There were no clinically relevant differences between devices in terms of hands-off time (Corpuls CPR 2.9% (1.1), LUCAS 3 3.7% (1.3), Easy Pulse 2.9% (0.7)), but a minimal statistically significant difference between LUCAS 3 and Easy Pulse (absolute difference 0.8% (0.2 to 1.5)).

We found significant differences regarding the proportion of effective compressions (Corpuls CPR 94% (7), LUCAS 3 98% (3), Easy Pulse 7% (4)) between all devices.

The difference between Corpuls CPR and LUCAS 3 was however minimal (− 3% (− 6.5 to − 0.3%)), whereas the difference between Easy Pulse and both Corpuls CPR (87% (84 to 91%)) and LUCAS 3 (91% (89 to 92%)) was considerable. Mean compression rate of Corpuls CPR (111 bpm; SD 1) was slightly higher than those of LUCAS 3 (104 bpm; SD 2) and Easy Pulse (102 bpm; SD 2).

We found no significant differences between devices for deviation of compression point. See Table 2 for an overview of all outcomes.

**Table 2:** Primary and secondary outcomes

Outcome	LUCAS 3	Corpuls CPR	Schiller EasyPulse	Δ LUCAS 3 vs Corpuls CPR (mean, 95% CI)	Δ LUCAS 3 vs Schiller EasyPulse (mean, 95% CI)	Δ Corpuls CPR vs Schiller EasyPulse (mean, 95% CI)
Performed work, mm (mean, SD)	65,877 (6163)	66,062 (2832)	40,177 (4396)	185 (−3346 – 3716)	25,700 (21,118 – 30,282)	25,885 (23,590 – 28,181)
Hands-off-fraction, % (SD)	3.7 (1.3)	2.9 (1.1)	2.9 (0.7)	−0.8 (−1.6–0.05)	0.8 (0.2–1.5)	0.1 (− 0.6–0.7)
Deep enough compressions, % (mean, SD)	98 (3)	94 (7)	7 (4)	−3 (− 6 – − 0.3)	90 (89–92)	87 (84–91)
Average compression depth, mm (mean, SD)	57 (2)	52 (1)	34 (4)	−5 (−6 – −4)	23 (21–25)	18 (17–20)
Average compression rate, bpm (mean, SD)	104 (2)	111 (1)	102 (2)	−7 (−6 – − 7)	1 (1–2)	8 (8–9)
Dislocation compression point, mm (mean, SD)	4 (2)	5 (3)	8 (6)	0.1 (−0.1–0.2)	−0.3 (− 0.7 – − 0.01)	−0.3 (− 0.6–0.1)

CI: confidence interval, SD: standard deviation

**Fig. 4:**
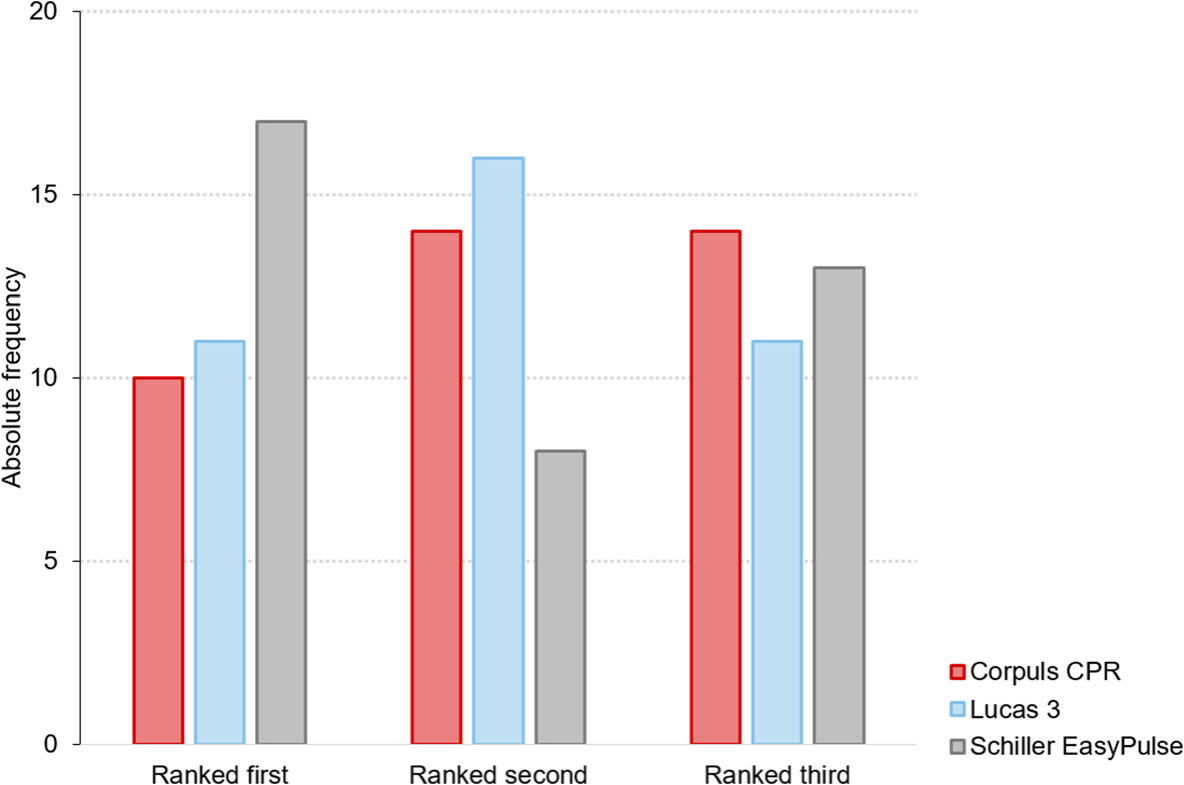
Personal ranking of devices

Noteworthy, despite the least favourable outcomes regarding objective measures of CPR quality, the Schiller Easy Pulse was ranked highest for personal preference by participants (see Fig. 4).

## Discussion

The study compared three mechanical chest compression devices showing the usability in an alpine rescue scenario.

Regarding our primary outcome performed work as a cumulative marker of quality of mechanical chest compressions, we found distinctly better results for both Corpuls CPR and LUCAS 3 than for Easy Pulse, with no significant differences between the former two devices. One reason for this could be the different compression technologies: A simple piston versus piston/band-combination.

Similar results were found regarding the secondary outcome of proportion of effective chest compressions. No significant differences in other secondary outcomes were recorded.

Up to date, there are no published studies on clinical outcomes of resuscitation with the Easy Pulse device in a human collective, therefore we can only speculate whether our findings regarding this device are just a matter of measurement or do really correspond to decreased quality of CPR.

Our findings however do support the assumption that both Corpuls CPR and LUCAS 3 are able to maintain high quality chest compressions even in rough alpine terrain.

We found that even during transport over steep and rough alpine terrain, the devices performed without any clinically relevant displacement. We nevertheless recommend marking the piston position after placement, and repeatedly control it during transportation to assure patient safety.

Our findings have severe influence on the management of cardiac arrest in the alpine setting. This is the first study to compare three different mechanical chest compression devices in a realistic alpine rescue scenario. Guidelines for cardiopulmonary resuscitation only permit intermittent CPR in case of hypothermic cardiac arrest, but these are very rare cases in alpine environment. Cardiac arrest of all other origins requires continuous chest compressions with minimal interruption. During transportation in an alpine setting, this seems to be only feasible using mechanical chest compression devices. Feasibility of this has been shown previously in the urban/flat rural setting [12]. The findings in our current study suggest that high quality mechanical chest compressions during an alpine rescue mission are achievable. This warrants the provision of such devices in HEMS and alpine rescue organizations.

Our results complement the findings of one previous study, in which mechanical CPR in alpine terrain was investigated. Thomassen et al. [12] report high quality of both manual and mechanical chest compressions during transportation on a sledge. Compared to our study, the scenario of this study was however much less representative for the reality of alpine rescue organizations, as mentioned before. Still, the findings of our study must be interpreted in light of the limitations of its design. This was a manikin study, and further research is needed to validate the applicability of findings in real patients. Further studies should also include ventilation as part of the scenario. Furthermore, we were not able to study one commonly used device, the Zoll AutoPulse, as mentioned above. The findings of our study might however be helpful to design and adequately power a study comparing the AutoPulse to the other devices. Design of such a study should especially consider the large differences found between the EasyPulse (using a combined piston & band system) and the other two devices, which used a conventional piston system only.

## Conclusion

Mechanical chest compression devices may provide a viable option for CPR in the alpine setting. Even if the use of such devices is expected to be generally rare in this setting, we found that short hand-off times and correct placement can be achieved, even by less trained personnel.

We could show that the Corpuls CPR and LUCAS 3 devices provided high quality chest compressions throughout the rescue scenarios. These devices also maintained correct placement of the piston even during challenging terrestrial transport. Further studies on the use of the Easy Pulse device are needed.

## Data Availability

The dataset used and/or analysed during the current study are available from the corresponding author on reasonable request.

## Abbreviations

BLS: Basic Life Support
CI: confidence interval
CPR: cardiopulmonary resuscitation
EMS: emergency medical service
EMT: emergency medical technician
SD: standard deviation
